# A holistic genome dataset of bacteria, archaea and viruses of the Pearl River estuary

**DOI:** 10.1038/s41597-022-01153-4

**Published:** 2022-02-14

**Authors:** Bu Xu, Fuyan Li, Lanlan Cai, Rui Zhang, Lu Fan, Chuanlun Zhang

**Affiliations:** 1 School of Environment, Harbin Institute of Technology, Harbin, China; 2 Shenzhen Key Laboratory of Marine Archaea Geo-Omics, Department of Ocean Science and Engineering, Southern University of Science and Technology (SUSTech), Shenzhen, China; 3 Daniel K. Inouye Center for Microbial Oceanography: Research and Education (C-MORE), University of Hawaii, Honolulu, Hawaii USA; 4 Department of Ocean Science, The Hong Kong University of Science and Technology, Hong Kong, China; 5 State Key Laboratory of Marine Environmental Science, Fujian Key Laboratory of Marine Carbon Sequestration, College of Ocean and Earth Sciences, Xiamen University, Xiamen, Fujian China; 6 Southern Marine Science and Engineering Guangdong Laboratory (Zhuhai), Zhuhai, China; 7 Southern Marine Science and Engineering Guangdong Laboratory (Guangzhou), Guangzhou, China

**Keywords:** Water microbiology, Metagenomics

## Abstract

Estuaries are one of the most important coastal ecosystems. While microbiomes and viromes have been separately investigated in some estuaries, few studies holistically deciphered the genomes and connections of viruses and their microbial hosts along an estuarine salinity gradient. Here we applied deep metagenomic sequencing on microbial and viral communities in surface waters of the Pearl River estuary, one of China’s largest estuaries with strong anthropogenic impacts. Overall, 1,205 non-redundant prokaryotic genomes with ≥50% completeness and ≤10% contamination, and 78,502 non-redundant viral-like genomes were generated from samples of three size fractions and five salinity levels. Phylogenomic analysis and taxonomy classification show that majority of these estuarine prokaryotic and viral genomes are novel at species level according to public databases. Potential connections between the microbial and viral populations were further investigated by host-virus matching. These combined microbial and viral genomes provide an important complement of global marine genome datasets and should greatly facilitate our understanding of microbe-virus interactions, evolution and their implications in estuarine ecosystems.

## Abstract (summary)

**Table:** 

Measurement(s)	bacteria • Archaea • viruses
Technology Type(s)	Shotgun Sequencing
Sample Characteristic - Organism	estuary metagenome
Sample Characteristic - Environment	subtropical estuarine
Sample Characteristic - Location	Pearl river estuary

Machine-accessible metadata file describing the reported data: 10.6084/m9.figshare.17139998

## Background & Summary

Estuaries are transitional environments between ocean and river. Complex and dynamic estuarine ecosystems are distinguishable from oceanic environments by significant variety of physical, chemical and geomorphologic conditions^1–4^. These factors have structured a highly unique estuarine microbial and viral community^5–7^. In addition, most estuarine ecosystems are impacted by strong anthropogenic stresses^1^. Viruses play essential roles in marine ecosystems by mortality^8,9^ and reprogramming the metabolic processes of hosts^10^. There is a great interest to investigate the genomic characteristics, evolutionary mechanisms, community composition and interactions of microorganisms and viruses in coastal environments^11,12^. While the abundance, distribution and function of prokaryotes or viruses in estuaries have been reported by using meta-omics approaches^13–18^, few studies have investigated bacteria, archaea and viruses simultaneously and none has delineated the potential connections between the microbiome and the virome. Therefore, a holistic estuarine genome dataset recovering both microbiome and virome will allow the analysis of microbe-virus interactions in this unique ecosystem.

The Pearl River is the second largest river in China with an average annual discharge flux of about 3.5 × 10^11^ m^3^ fresh water and 8.87 × 10^7^ tons suspended sediment^19^. Locating in the most densely industrialized and urbanized region in China, the Pearl River is heavily impacted by human activities including agricultural irrigation, industrial and domestic emissions and aquaculture^20,21^. While some ecological and genomic studies on the bacterial or viral communities at the Pearl River estuary (PRE) have been performed^13,15,17^, none of them has produced a combined dataset including both the microbial hosts and the viruses. Such a dataset is therefore urgently demanded to unveil the dynamic and diverse biological processes coupling with physiochemical factors at this estuary.

**Fig. 1:**
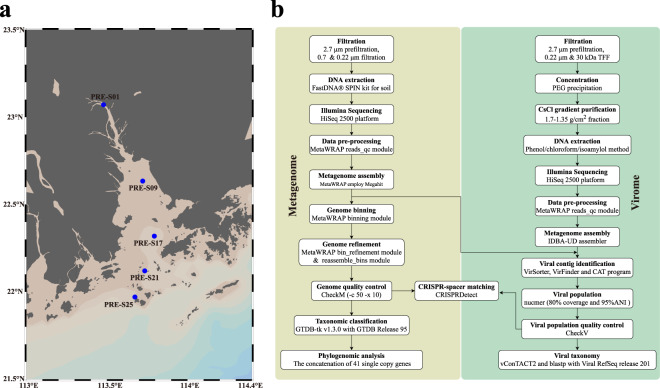
Sampling sites in the PRE and methods used for this study. (**a**) yellow dots represent the sampling sites. (**b**) the study workflow in processing PRE metagenome sequences.

Here, we sequenced 15 deep-sequencing metagenomes of surface water with three size-fractions collected at five sampling sites along the salinity gradient of the PRE in August 2016 (Fig. 1a). Seawater was filtered through cellulose membranes subsequently. The 0.7–2.7 μm and 0.22–0.7 μm fractions were used to produce particle-attached and free-living prokaryotic metagenomes, respectively. To collect the viral fraction, surface water was prefiltered by using filters of 2.7 μm and 0.22 µm pore-size, subsequently, and then concentrated with 30 kilodalton (kDa) pore-size filters by using tangential-flow filtration. Further concentration and purification were done via polyethylene glycol (PEG) precipitation and cesium chloride (CsCl) step-gradient ultracentrifugation (Fig. 1b). DNA was extracted from the cellular (0.7–2.7 μm and 0.22–0.7 μm) and viral (<0.22 μm) fractions for metagenomic sequencing.

**Fig. 2:**
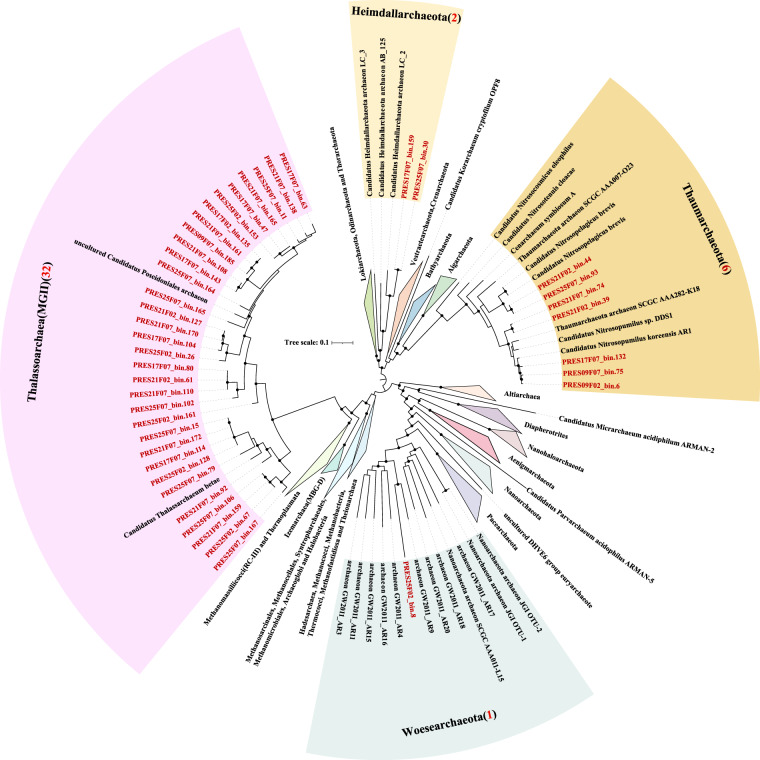
Phylogenomic analysis of archaeal MAGs. The maximum likelihood tree was reconstructed based on the concatenation of 41 single copy marker genes spanning a set of 41 MAGs (in red) obtained in this study and a set of 163 reference genomes (in black). The number of MAGs discovered in this study in each phylum is indicated in the parenthesis after the phylum name. The bootstrap values >0.9 are shown as dots on nodes. The tree is unrooted. Source data are provided as a Source Data file.

**Fig. 3:**
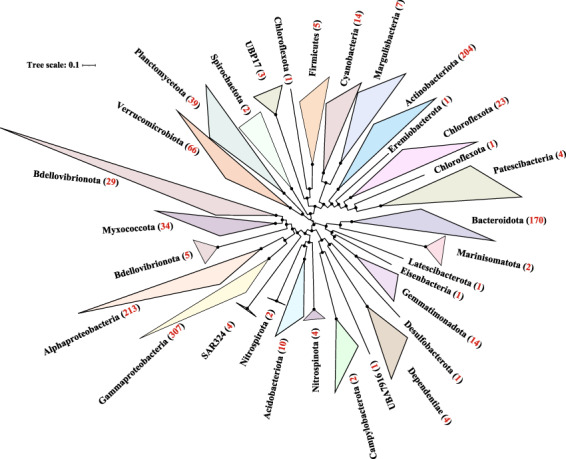
Phylogenomic analysis of bacterial MAGs. The maximum likelihood tree was reconstructed based on the concatenation of 41 single copy markers. The number of MAGs discovered in this study in each phylum is indicated in the parenthesis after the phylum name. Number of MAGs from the PRE metagenomes in each phylum or class are indicated in between parenthesis in red. The bootstrap values >0.9 are shown as dots on nodes. The tree is unrooted. Source data are provided as a Source Data file.

**Fig. 4:**
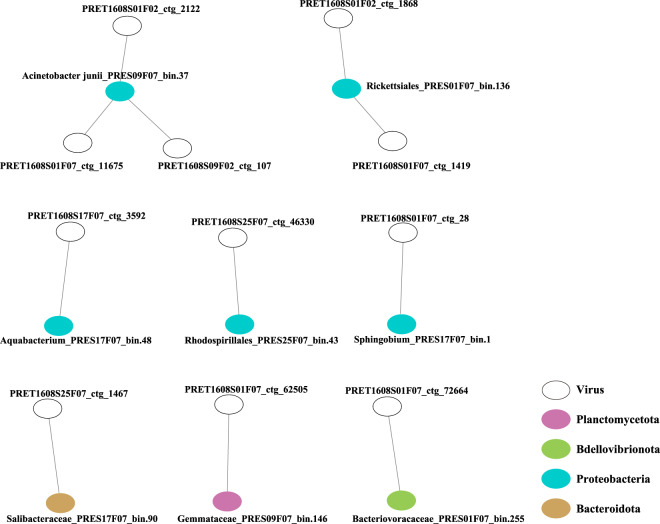
Network analysis of virus-host pairs. The hollow circles represent the viruses. The solid circles represent the prokaryotic hosts. The colors indicate the phyla of the hosts.

Overall, 13,305,017 contigs were generated by assembling quality checked sequencing reads (Table 1). A total of 1,205 non-redundant metagenome assembled genomes (MAGs) with the estimated completeness ≥50% and contamination ≤10% were reconstructed based on multi-strategy binning according to the MIMAG criteria^22^ (Supplementary Table 1). Phylogenomic analysis based on single-copy marker genes showed that these MAGs belonged to 32 bacterial and four archaeal phyla according to the Genome Taxonomy Database (GTDB) taxonomy^23^ (Fig. 2, 3). We found that 24.8% and 86.8% of total MAGs did not have close relatives at genus and species level based on 95% average nucleotide identity (ANI). A total of 78,502 non-redundant viral contigs were predicted from the cellular microbiomes (0.2–2.7 μm) and viromes (<0.2 μm). They were then clustered into 56,289 viral populations^24–26^. Taxonomic classification of viral populations was performed based on closest relative affiliation^24^ (Supplementary Table 2). Only 15.3% populations could be assigned according to the RefSeqVirus database leaving the rest majority unclassified. A total of 15 viral families were identified including ssDNA, dsDNA and ssRNA viruses and the primary group belongs to order *Caudovirales* (Table 2). Virus-host pair prediction was performed based on clustered regularly interspaced short palindromic repeats (CRISPR) -spacer matching and 11 virus-host pairs were identified (Fig. 4). Among them, an *Acinetobacter junii* and a Rickettsiales bacterium were found being infected by more than one type of virus.

**Table 1:** Summary of reads, contigs, MAGs and viral contigs of PRE metagenomes.

Station	Size-fraction	Raw read pairs	Read pairs after QC	Contigs (>1 kb)	Prokaryotic MAGs*	Viral contigs (>5 kb)
PRE-S01	0.7–2.7 μm	442,832,413	249,009,883	1,497,910	256	7,539
	0.22–0.7 μm	408,434,124	297,683,700	1,155,059	178	9,479
	<0.22 μm	81,475,964	66,065,092	114,587	Not Applicable	1,373
PRE-S09	0.7–2.7 μm	449,343,573	320,412,019	1,330,764	239	5,027
	0.22–0.7 μm	477,408,408	299,346,960	1,300,578	204	10,069
	<0.22 μm	18,803,045	15,540,200	26,853	Not Applicable	430
PRE-S17	0.7–2.7 μm	461,596,030	322,152,520	920,756	191	3,316
	0.22–0.7 μm	472,049,471	312,545,342	702,047	134	9,404
	<0.22 μm	22,655,869	18,478,415	31,626	Not Applicable	1,043
PRE-S21	0.7–2.7 μm	462,296,138	319,291,227	945,976	182	5,025
	0.22–0.7 μm	475,167,759	306,620,589	929,998	143	16,029
	<0.22 μm	21,784,687	17,794,588	48,363	Not Applicable	1,919
PRE-S25	0.7–2.7 μm	462,183,037	292,078,554	1,160,660	182	6,963
	0.22–0.7 μm	467,795,025	295,708,591	1,001,020	169	12,710
	<0.22 μm	19,058,316	15,808,251	48,012	Not Applicable	2,258

*Completeness >50%, contamination <10%.

**Table 2:** Nonredundant contigs of abundant viral populations in samples.

Major Taxa	PRES01V*	PRES01FL**	PRES01PA***	PRES09V	PRES09FL	PRES09PA	PRES17V	PRES17FL	PRES17PA	PRES21V	PRES21FL	PRES21PA	PRES25V	PRES25FL	PRES25PA
Unassigned	1,301	8,223	6,572	398	8,620	4,464	967	7,582	2,821	1,765	13,416	4,280	2,086	10,195	5,715
Myoviridae	9	933	712	4	1,207	459	17	1,512	314	76	1,926	520	76	1,840	833
Siphoviridae	29	159	105	18	117	55	19	106	93	23	167	75	20	151	106
Podoviridae	24	73	56	10	45	14	22	74	20	50	233	46	63	170	63
Autographiviridae	1	7	6	0	11	2	18	53	22	5	134	37	11	198	168
Phycodnaviridae	0	36	32	0	37	12	0	50	32	0	97	45	2	111	54
Demerecviridae	0	31	31	0	25	17	0	22	6	0	37	12	0	32	12
Mimiviridae	0	12	23	0	5	2	0	1	1	0	12	7	0	5	2
Iridoviridae	0	1	0	0	0	1	0	1	0	0	3	1	0	3	5
Herelleviridae	1	2	1	0	2	1	0	3	0	0	2	1	0	2	0
Microviridae	0	0	0	0	0	0	0	0	6	0	0	1	0	2	2
Lavidaviridae	8	0	0	0	0	0	0	0	0	0	0	0	0	0	1
Inoviridae	0	1	1	0	0	0	0	0	1	0	1	0	0	1	1
Poxviridae	0	0	0	0	0	0	0	0	0	0	1	0	0	0	0
Metaviridae	0	0	0	0	0	0	0	0	0	0	0	0	0	0	1
Marseilleviridae	0	1	0	0	0	0	0	0	0	0	0	0	0	0	0

*V, viral fraction (siz <0.22 μm);

**FL, free-living cellular fraction (size 0.22–0.7 μm);

***PA, particulate-associated cellular fraction (size 0.7–2.7 μm).

All of the primary contigs, non-redundant MAGs and viral-like contigs have been deposited in the National Center for Biotechnology Information (NCBI) BioProject database and the *figshare* website. The microbial and viral genomes provided here suggest great biological diversity in the PRE ecosystems. This combined dataset allows for systematic study on microbial-virial interactions including the regulatory mechanisms of viruses in manipulating estuarine biogeochemistry under anthropogenic impacts.

## Methods

### Sampling, DNA extraction and sequencing

Bacterial, archaeal and viral sample collection and particle size-based fractionation was done by filtration^27^. To obtain the cellular fractions, about 500 L surface water (0.5–1.0 m in depth) was collected at each sampling site in PRE in August 2016 within three days (Fig. 1a, Table 3). The water samples were first filtered through 2.7 μm pore-size glass fiber filters (Shanghai Mosutech, Shanghai, China) to remove large particles and the filtrates were then successively filtered through 0.7 and 0.22 μm pore-size membrane filters (Pellicon cartridge, Millipore Corp., Billerica, MA, USA) to collect particulate associated and free-living microbial cells, respectively. The filters were stored in liquid nitrogen temporarily on board and then transferred to −80 °C freezers when back to laboratory for long-term storage until further processing. To collect viral particles, 200 L prefiltered seawater was further filtered through 2.7 μm and 0.2 μm pore-size membrane filters. A tangential-flow filtration 30 kDa cartridge was (0.5 m^2^ Pellicon cartridge, Millipore Corp., Billerica, MA, USA) applied to increase viral particle concentration till a final liquid volume of 2 L and the liquid was kept at 4 °C till further process^28^. Physiochemical measurements of water and the methods to generate these measurements have been published by He *et al*.^23^. The measurements are also available in Table 3.

**Table 3:** Sampling locations and bulk properties of PRE surface water.

Station	PRE-S01	PRE-S09	PRE-S17	PRE-S21	PRE-S25
Latitude (°N)	23.0717	22.634742	22.319517	22.120133	21.9717
Longitude (°E)	113.479733	113.722569	113.795633	113.735033	113.67375
Sampling time	2016.08.23 12:50	2016.08.22 12:28	2016.08.21 15:58	2016.08.21 11:21	2016.08.20 12:00
Temperature (°C)	30.8	31.5	28.6	27.3	27.4
Salinity (PSU)	0.12	1.17	11.24	22.21	28.05
pH	6.5	6.77	7.42	7.83	8.06
DO (μM)	39.38	123.16	138.75	138.44	147.81
DOC (μM)	182.67	137.5	113.08	99	78.58
TDN (μM)	361.29	197.36	165.71	87.64	39.79
NO^3-^ (μM)	94.69	132.12	82.55	32.17	28.9
NO^2-^ (μM)	23.49	1.628	19.09	11.7	9.5

DNA was extracted from the 0.2 and 0.7 μm pore-size membrane filters by using the FastDNA® SPIN kit for soil (MP Biomedicals, Solon, OH, USA) following the manufacturers’ instructions. For virome samples, a series of enrichment operations were applied to increase the concentration of the virial suspension^28^ (Fig. 1b). Firstly, PEG8000 (10% w/v) was dissolved in DNase I (Sigma-Aldrich) treated viral concentrate and incubated at 4 °C overnight to precipitate viral particles. The PEG pellet was resuspended after centrifugation (10, 000 × g for 1 h) and then purified by CsCl density gradient ultracentrifugation (1.7, 1.5, and 1.35 g/mL CsCl layers). After centrifugation, viral like particles was concentrated in 1.5–1.35 g/mL CsCl layers according to the physical properties of various virions. After collection and purification, a phenol-chloroform extraction following the ethanol precipitation method was applied to extract viral genomic DNA^14,28^.

The extracted prokaryotic and viral DNA were fragmented by sonication to a size of 350 bp. The DNA fragments were then end-polished, A-tailed, and ligated with the full-length adaptor to construct TruSeq metagenome libraries. Libraries were analyzed for size distribution using the Agilent2100 Bioanalyzer (Agilent, USA) and quantified using real-time PCR. They were then sequenced on an Illumina HiSeq 2500 platform at Novogene Bioinformatics Technology Co., Ltd. (Beijing, China) to generate 150 bp paired-end reads. The FASTQ files containing raw reads are available on NCBI. The overall study workflow is show in Fig. 1b.

### Sequence quality check and assembly

The reads_qc module of MetaWRAP (v1.2.1)^29^ was applied for adaptor trimming and contamination removal for the raw sequencing reads to generate high-quality clean reads by calling Cutadapt^30^ and FastQC^31^ with the default parameters. Clean reads of the cellular fractions were assembled into contigs by using MetaWRAP employing megahit with k-mer values list of 21, 29, 39, 59, 79, 99, 119 and 141^29^. The IDBA-UD software (v1.1.3) was applied to assemble the viral metagenomes with default parameters^32^. Contigs of length longer than 1 kb were used for further analysis as suggested by the MIMAG and the MIUViG standards^22,26^ (Table 1).

### MAG generation, refinement, quality check and taxonomic annotation

For each prokaryotic metagenome, MAGs were recovered by using the binning module and bin_refinement module of MetaWRAP^29^. First, the binning module of MetaWRAP employing METABAT^33^ and CONCOCT^34^ was applied to recover the original genome MAGs sets based on tetranucleotide frequencies and read coverage. These MAGs sets were pooled and dRep (v2.6.2) was performed to remove redundant MAGs^35^. The bin_refinement module of MetaWRAP was used to refine the MAGs to produce final MAGs. The completeness and contamination of archaeal and bacterial MAGs were estimated by running CheckM (v1.0.11)^36^ (Supplementary Table 1). Taxonomic classification of the final MAGs was conducted by using GTDB-tk (v1.3.0, Release 95)^37^ (Supplementary Table 1). MAGs are considered of the same species if they have ANI values larger than 95% by compared to a reference genome.

### Phylogenomic analysis

We used 41 single-copy marker proteins to infer the maximum likelihood trees of archaeal and bacterial MAGs^38,39^, respectively. Specifically, putative coding DNA sequences for each draft genome were predicted by using Prodigal (v2.6.3; -m -p meta)^40^. Putative single copy genes of each MAGs were identified by using hmmsearch (HMMER v.3.1b2; -E 1E-5)^41^ based on Hidden Markov Models (HMMS) described by Sunagawa *et al*.^39^. Amino acid sequences of these genes were aligned, respectively, by using Clustal Omega (v1.2.4)^42^ and further automatically trimmed by using trimAL (v1.4.1; -automated1)^43^. The alignments of proteins were concatenated by using ScaFos (v1.2.5) and missing data were filled with gaps^44^. The phylogenomic tree of concatenated alignment was reconstructed by using IQ-TREE (v.2.0.3; -st AA -m LG + PMSF + G -B 1000 --bnni)^45^ and visualized in the Interactive Tree of Life (iTOL, v.5.1.1)^46^.

### Viral contig identification, dereplication and taxonomic classification

Following assembly, putative viral contigs were identified from contigs of all the three size fractions with length greater than 1.5 kb by using VirSorter (v1.0.6)^47^ and VirFinder (v1.1)^48^ as described by Gregory *et al*.^24^. First, contigs identified as ‘lytic/prophage categories 1 and 2′ and ‘circular’ by VirSorter were assigned as viral contigs. The rest contigs of length >5 kb were kept for further classification. Among them, those identified as ‘lytic/prophage categories 1,2′ by VirSorter, or as viruses by VirFinder with score >0.9 (p < 0.05) were assigned as viral contigs. Those identified as ‘lytic/prophage category 3′ by VirSorter and as viruses by VirFinder with score 0.7–0.9 (p < 0.05) were also assigned as viral contigs. Those identified as ‘lytic/prophage category 3’ by VirSorter but not as viruses by VirFinder with score >0.7 (p < 0.05), and those identified as viruses by VirFinder with score 0.7–0.9 (p < 0.05) but not as ‘lytic/prophage categories 1–3’ by VirSorter were further analyzed through CAT^49^ and only those having 40% genes classified as viruses were kept. In total, 97,003 viral contigs were identified. Redundancy of these contig sequences was removed by using CD-HIT at 99% identity (v4.6.8, −c 0.99 −aS 0.99)^50^. The resulting 78,502 non-redundant viral contigs were further grouped into 56,289 viral populations by using nucmer based on the criterion that virial contigs in the same population share 80% of their genes and have 95% average nucleotide identify as previously described^51,52^ (Fig. 1b). CheckV (v0.8.1) was used to determine the completeness and quality of the identified viral populations^53^ (Supplementary Table 3). We used VirSorter to identify prophages by the *de novo* predictions of categories 4 and 5^47^.

Taxonomic classification of viral populations was performed with a complementary approach by using vConTACT2^54^ and blastp^55^. First, the ORFs of each population were derived by using prodigal^40^. Second, the protein sequences of population contigs >10 kb were analyzed by using vConTACT2 with Viral RefSeq release 201 based on genome gene-sharing profiles. Then, family level taxonomy of the remaining population including those that could not be assigned by vConTACT2 were further defined by closest relative affiliation using blastp against the Viral RefSeq database with the following principle: identity ≥30%, bit-score ≥50, and E value ≤0.001. Only the population with more than half of proteins assigned to the same viral family was considered as a viral family (Supplementary Table 2).

### Host prediction of viral sequences

In order to link viral contigs to their putative microbial hosts, CRISPR spacers in MAGs were identified by using CRISPRDetect (v2.5)^56^. Spacer sequences were then matched to viral contigs by using fuzznuc^57^. Host and virus infection networks were reconstructed in Cytoscape (v3.8.0)^58^.

## Data Records

Raw reads generated in this study have been deposited in the National Center for Biotechnology Information BioProject database with the project ID PRJNA763043^59^. Contigs, MAGs, viral genomes and source data files including the genome trees and associated amino acid alignments have been deposited in the *figshare* website^60^. A full copy of this dataset is also available in the National Omics Data Encyclopedia (https://www.biosino.org/node/) with the project ID OEP001662^61^.

## Technical Validation

Additional technical validation should be applied by researchers to confirm the accuracy of draft MAGs and VAGs used for specific downstream purposes.

## Supplementary information

Supplementary Table 1

Supplementary Table 2

Supplementary Table 3

## Data Availability

All versions of third-party software and scripts used in this study are described and referenced accordingly in the Methods sub-sections for ease of access and reproducibility.
